# Acute tibialis posterior and flexor digitorum longus tendons rupture after glass laceration: A rare case report

**DOI:** 10.1016/j.radcr.2026.05.020

**Published:** 2026-06-08

**Authors:** Aliasghar Alamian, Parmida Shirzadi, Mohammad Ali Shirazi, Amirhossein Amiri

**Affiliations:** a Bone Joint and Related Tissues Research Center, Akhtar Orthopedic Hospital, Shahid Beheshti University of Medical Sciences, Tehran, IR Iran; b Student Research Committee, School of Medicine, Shahid Beheshti University of Medical Sciences, Tehran, Iran

**Keywords:** Tibialis posterior tendon, Flexor digitorum longus, Tendon laceration, Magnetic resonance imaging, Ankle trauma, Case report

## Abstract

Acute posterior tibialis tendon rupture is a rare injury, most often occurring in association with ankle fractures or severe sprains, and isolated traumatic lacerations are exceptionally uncommon. In this study, we present a 53-year-old Iranian female who arrived with persistent medial ankle pain and lameness as her chief complaint following a glass-induced laceration. On physical examination, she exhibited tenderness and swelling along the medial ankle, difficulty performing a single heel-rise, and weakness in foot inversion, while neurovascular status remained intact. MRI evaluation demonstrated complete rupture of both the posterior tibialis and flexor digitorum longus tendons, without any concomitant ankle fractures. Intraoperative findings confirmed the diagnosis, and given the condition of the tendon ends, primary tendon repair was selected as the surgical approach. The ankle was immobilized for six weeks postoperatively, after which physiotherapy was initiated. At the three-month postoperative follow-up, the patient demonstrated significant pain relief, improved inversion strength, and normalization of gait.

## Introduction

The tibialis posterior tendon functions as the primary dynamic stabilizer of the medial arch, playing a crucial role during the stance phase by facilitating inversion, adduction, and plantar flexion of the foot [1,2]. Its rupture, although uncommon [3], is often either discovered during surgery or overlooked when the initial clinical assessment is limited [4,5]. Typically, patients experiencing dysfunction of this tendon exhibit signs such as diminished strength, discomfort along the tendon pathway, swelling around the medial malleolus, and varying degrees of arch reduction or onset of flatfoot prior to complete rupture [6]. Clinical suspicion should be heightened in individuals presenting with persistent medial ankle pain and swelling, especially following a twisting mechanism. The physical examination frequently reveals a flexible flattening of the foot (asymmetric pes planus), forefoot pronation, and lack of normal tibialis posterior activity. In these cases, patients are unable to perform a single-leg heel raise on the affected side, while the opposite foot usually displays a less pronounced flattening [4]. Failure to identify ruptures of the tibialis posterior tendon may lead to progressive, painful flatfoot deformity, predominantly due to the unopposed action of the peroneus brevis and insufficient support of the medial longitudinal arch [5].

The present report discusses a unique case involving simultaneous tibialis posterior and flexor digitorum longus tendons laceration caused by a glass injury, complicated by delayed diagnosis and management. The absence of a medial malleolus fracture and the combined rupture illustrate the clinical rarity and underscore the value of this case, representing the first documented instance of both tendons being severed in a glass trauma scenario.

## Case presentation

A 59-year-old female presented to our orthopedic clinic with chronic medial left ankle pain and progressive antalgic gait (lameness), occurring approximately 7 weeks (53 days) post-traumatic laceration to the medial aspect of her left ankle from broken glass during a household accident. The initial injury was managed at an urgent care facility with basic saline irrigation (volume unspecified) and primary closure using interrupted sutures, without the use of imaging, tetanus prophylaxis, or assessment for underlying tendinous, neurovascular, or osseous damage. Over the subsequent 4-6 weeks, her condition progressed insidiously, characterized by dynamic medial instability, impaired stair negotiation, and intolerance to standing for more than 10-15 minutes. Her past medical history was significant only for controlled essential hypertension and hypothyroidism.

Physical exam revealed 3-4 cm laceration scar along the medial ankle, posterior to the medial malleolus, without erythema, induration, or discharge. Palpation demonstrated focal tenderness over the tibialis posterior tendon sheath; the flexor digitorum longus sheath showed mild tenderness as well. The patient was unable to perform a single-leg heel rise on the left side, with compensatory subtalar eversion and mild flexible planovalgus foot alignment observed during weight-bearing stance. Active and passive range of motion at the ankle and subtalar joint eversion was preserved and symmetric bilaterally. Other neurovascular examination was normal in sensation. In vessels examination, Dorsalis pedis and posterior tibialis arteries puses were detectible with normal capilliary filling in toes. No signs of infection, wound dehiscence, or deep vein thrombosis were found.

Magnetic resonance imaging (MRI) of the left ankle revealed complete laceration of both the tibialis posterior tendon and the flexor digitorum longus tendon, with retraction of the tendon ends to proximal side. Soft-tissue edema was obvious as well, but no bony injury, fracture, or additional tendon damage was observed (Fig. 1).

**Fig. 1:**
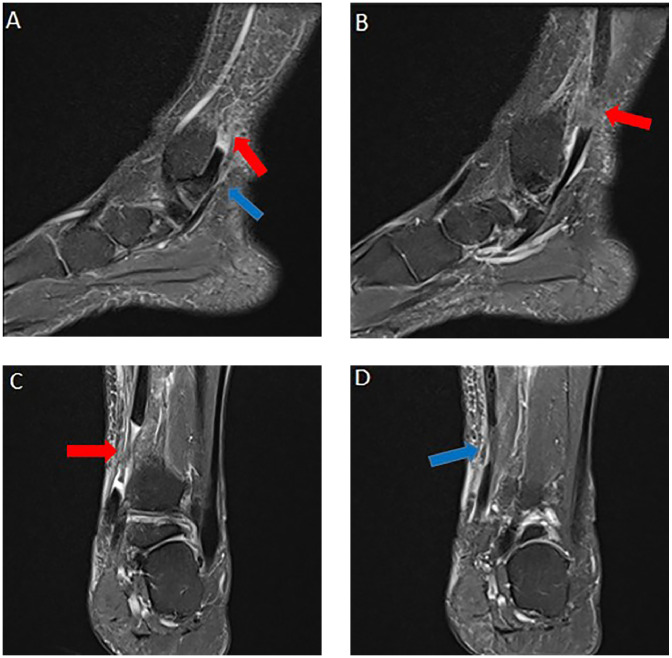
Diagnostic patient left ankle MRI, (A) Sagittal T2-weighted MR image of the left foot and anckle reveals discontinuity, retraction and increased signal intensity at the course of the tibialis posterior (red arrow) and flexor digitorum longus (blue arrow) tendons, indicative of complete tendon rupture. (B) A fluid-filled gap and surrounding soft tissue edema are evident at the injury site, consistent with delayed presentation following penetrating trauma (red arrow). (C, D) Coronal T2-weighted MRI providing further detail of the tendon rupture in the left ankle, with a visible gap and signal changes demonstrating a complete rupture of both the tibialis posterior (red arrow) and flexor digitorum longus (blue arrow) tendons in the left ankle.

Given the delayed presentation and functional deterioration, surgical exploration and repair were recommended. Intraoperatively, both tendons were found to be sharply transected, with moderate retraction, but with preserved tissue quality. Because the tendon ends were visible without extreme gap, primary end-to-end repair of both the tibialis posterior and flexor digitorum longus tendons was performed using nonabsorbable core sutures reinforced with epitendinous stitches (Fig. 2).

**Fig. 2:**
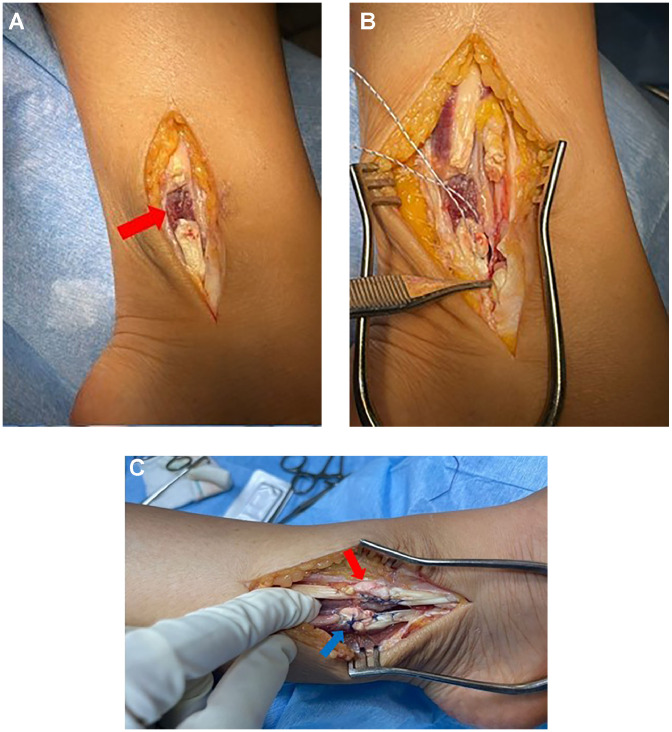
Intraoperative findings. (A) Surgical exposure of the medial ankle showing the Posterior tibialis rupture (blue arrow). (B) Identification of the retracted posterior tibialis and flexor digitorum longus tendon ends. (C) Performed surgical repair for both tibialis posterior (red arrow) and flexor digitorum longus (blue arrow) ruptured tendons.

Postoperatively, the ankle was immobilized by short leg casting in plantarflexion and inversion positin for six weeks, with non-weight-bearing instructions. Left ankle physiotherapy, emphasizing restoration of ankle mobility with muscle strengthening was initiated for patient after six weeks. At the three-month follow-up visit, the patient demonstrated significant improvement, with remarkable reduction in medial ankle pain and gait normalization. Additionally, she was able to initiate a partial single-heel raise, indicating favorable tendon function recovery. It is worthy to note, no complications were observed.

## Discussion

The posterior tibialis is the deepest muscle in the posterior compartment of the leg. It originates from the interosseous membrane and partially from the posterolateral surface of the tibia and the upper fibula. Just above the ankle, the tendon passes anterior to the flexor digitorum longus and then continues within a groove behind the medial malleolus, beneath the flexor retinaculum. In the hindfoot, it courses superficial to the deltoid ligament and deep to the plantar calcaneonavicular (spring) ligament, sending fibrous extensions before inserting primarily into the navicular and other tarsal bones, except the talus [7]. Functionally, the tendon initiates hindfoot inversion and contributes to formation of a rigid lever through the tarsal bones. Its dysfunction disrupts this mechanism and can result in acquired flatfoot deformity with progressive chronic pain [8].

Rupture of the posterior tibialis tendon (PTT) is most often associated with systemic diseases or comorbidities such as hypertension, long-standing diabetes, rheumatoid arthritis, connective tissue disorders, obesity, or advanced age [9,10]. Acute traumatic PTT rupture is considered rare and has been described mainly in association with ankle fractures—particularly those involving the medial malleolus—or repeated ankle sprains in athletes. Megan et al. [8] reported 35 case reports of traumatic PTT rupture between 1980 and 2021, all associated with an ankle fracture. To the best of our knowledge, only a small number of studies [1,6,[11], [12], [13], [14]] have described acute PTT rupture without a medial malleolus fracture. Monto et al. [11] were the first to report such a case. Wardell et al. [12] later described a closed bimalleolar-equivalent ankle fracture without medial malleolus involvement. Martinelli et al. [6] and Rayes et al. [13] reported acute PTT rupture without any ankle fracture, with mechanisms of ankle sprain and crack jumping. In the study by Korovessis et al. [14], a distal tibial fracture caused by a motor-vehicle accident led to PTT rupture. A more recent report by Agrawal et al. [1] described a traumatic tibialis posterior tendon rupture caused by a grinder injury, also without any associated fracture. In contrast to these reports, our case involved an acute PTT rupture without any associated fracture, caused by a glass laceration—a mechanism not previously described. Additionally, our patient sustained simultaneous rupture of both the PTT and the flexor digitorum longus (FDL) tendon, a combination previously reported only once, by Korovessis et al. [14].

Regarding patient-specific factors, Martinelli et al. [6] and Korovessis et al. [14] reported no systemic risk factors in their cases, while Rayes et al. [13] described corticosteroid injection 15 days prior to injury. Similar to those cases, our patient had no diabetes, rheumatoid arthritis, or connective tissue disorder, although hypertension and hypothyroidism were present—conditions that some studies have suggested as possible contributors to PTT dysfunction [15].

Diagnosis of acute PTT rupture can be challenging because pain and swelling often limit physical examination. Tenderness along the medial malleolus and difficulty performing a single-heel raise may suggest PTT injury [16], and collapse of the medial longitudinal arch may reveal a positive “too many toes” sign [17]. Radiographs are useful for identifying associated fractures, and Stein [18] noted that metaphyseal bone flakes might be a clue to PTT rupture. Ultrasonography has been recommended as an initial tool for tendon evaluation, particularly when radiographs are normal [13], while MRI is more accurate in advanced or complex presentations [16]. Although ultrasonography is highly sensitive for detecting foreign bodies such as glass, MRI was selected in this case due to the delayed presentation and the need for comprehensive evaluation of soft tissue structures, particularly tendon integrity and the extent of retraction. MRI provided detailed anatomical information that was essential for surgical planning. Despite imaging, many studies have emphasized that definitive diagnosis is often confirmed only during surgery [13].

Once PTT rupture is recognized, early treatment is necessary to prevent the development of flatfoot deformity and secondary osteoarthritis [13]. In cases where there is a large tendon gap or segmental loss, tendon transfers such as FDL or flexor hallucis longus (FHL) may be considered. Although there is a size discrepancy between FDL and PTT, their anatomic proximity makes transfer feasible, and patients generally maintain satisfactory heel-rise ability after transfer [8]. When retraction is extensive, allografts may be required, as described by Cavaliere et al. [16] in a case with a 6-cm defect. El Rayes et al. [13] reported successful reconstruction with a hamstring tendon graft in a young athlete. Martinelli et al. [6] used a combination of medial calcaneal displacement osteotomy and FDL transfer due to severe tendon degeneration. Wardell et al. [12] performed primary repair with FDL transfer because of PTT dysfunction. Taken together, the size of the tendon gap plays an important role in determining the surgical approach. Small gaps with minimal retraction can be managed with primary end-to-end repair, whereas larger gaps or significant tendon loss may require tendon transfer or graft reconstruction. In our case, both the PTT and FDL tendons were sharply lacerated, and the tendon ends were healthy with no significant gap, allowing approximation of the tendon ends without tension. Therefore, successful primary suture repair of both tendons was considered appropriate in our case, similar to the case described by Korovessis et al. [14].

## Conclusion

This case demonstrates the unusual occurrence of delayed, combined tibialis posterior and flexor digitorum longus tendon lacerations resulting from a glass injury, with no accompanying fracture. Persistent medial ankle dysfunction and inability to perform a single-heel rise should heighten suspicion for deep tendon involvement and warrant prompt advanced imaging. Because tendon quality remained adequate, primary repair was achievable and followed by a structured rehabilitation program that restored strength and gait mechanics. This report highlights the critical role of early recognition and surgical intervention in preventing progression to fixed planovalgus deformity and long-term functional impairment.

## Patient consent

Written informed consent was obtained from the patient for publication of this case report and the accompanying images.
